# External assessment of medical education quality: indicative model development considering paradox of skill

**DOI:** 10.3389/fpubh.2023.1184861

**Published:** 2023-07-11

**Authors:** Artem Artyukhov, Beata Gavurova, Iurii Volk, Svitlana Bilan, Serhiy Lyeonov, Tawfik Mudarri

**Affiliations:** ^1^University of Economics in Bratislava, Bratislava, Slovakia; ^2^Sumy State University, Sumy, Ukraine; ^3^Department of Addictology, First Faculty of Medicine, Charles University and General Teaching Hospital in Prague, Prague, Czechia; ^4^Rzeszów Uniersity of Technology, Rzeszów, Poland; ^5^Silesian University of Technology, Gliwice, Poland; ^6^The London Academy of Science and Business, London, United Kingdom; ^7^Faculty of Mining, Ecology, Process Control and Geotechnologies, Technical University of Košice, Košice, Slovakia

**Keywords:** medical education, education quality, education funding, education evaluation, bibliometric analysis, sustainable development goals

## Abstract

This study proposes an approach to the external evaluation of medical education programs' quality based on a combination of indicators, including international rankings, external stakeholders' input, and independent agencies' assessments. We modify the success equation with a detailed consideration of the skill component and its decomposition into internal and external quality assurance elements along with authority. We carried out a bibliometric analysis regarding the problem of medical education quality assessment in the context of achieving sustainable development goals. We described the calculation model of external quality assessment indicators through the algorithms of independent education quality assurance agencies' activity and rating indicators shown in the modified Mauboussin's equation. The model considers the economic component (the consequence of achievement) of skill, which is expressed in raising funds from external sources to implement educational and scientific activities. The proposed algorithm for assessing the educational program quality can be applied to benchmark educational program components, complete educational programs within the subject area, and the educational institution for different areas. We propose a “financial” model for educational program quality based on the analysis results. The model makes it possible to determine the need for additional focused funding of the educational program based on the individual analysis of the external evaluation criteria of the achievement level. This study analyzes the accreditation results of more than 110 educational programs in 2020 and 8 months of 2021 within the direction 22 “Medicine” (according to the national classification of fields of knowledge) (state and private Ukrainian medical universities).

## 1. Introduction

The quality of medical education is a popular area of study by scientists in various applications:

- at the national level, with proposals for the development of strategies for improving educational programs (1);
- at the regional level, taking into account the specifics of medical programs (2, 3);
- in terms of institutional accreditation in general (4);
- communication with practitioners and employers (5, 6);
- discussions about the importance and influence of individual indicators on the quality of medical educational programs (7–9);
- the role of stakeholders in ensuring the quality of medical education (10, 11), etc.

As the analysis of the data presented above shows, assessing the quality of education (especially external, which is implemented by independent agencies) is carried out qualitatively (the presence or absence of indicators according to various criteria). The lack of a quantitative assessment complicates the evaluation process to some degree. A tool that can quantify at least some of the indicators is needed.

External accreditation of educational programs by the National Agency for Higher Education Quality Assurance is currently the only tool for assessing the quality of medical education in universities under the Ukrainian Ministry of Education and Science and the Ministry of Health.

State regulation of educational activities (12, 13) to meet the Sustainable Development Goals (14, 15) can only partially satisfy the requirements of educational services consumers. Innovation strategies (16, 17), providing leadership in the implementation of breakthrough technologies (18, 19), choosing the most effective forms of the educational process (20, 21), creating a successful socio-economic model of the university (22–24), promoting the university brand on the market of educational and scientific services (19, 25), evaluation of educational program quality by stakeholders (26, 27), and the openness of information about the educational program (28, 29)—all these characteristics are the subject of examination during the implementation of external education quality assessment.

This study demonstrates that quality assurance in education is a multifactorial “experiment”. All components of the socio-economic state of the region are essential, especially the organization of training in different periods, the influence of external factors on the demand for educational programs, etc. (30–52).

The research is based on the example of data analysis in the field of “Medicine”; the research results can be further applied to other educational areas.

**Figure 1:**
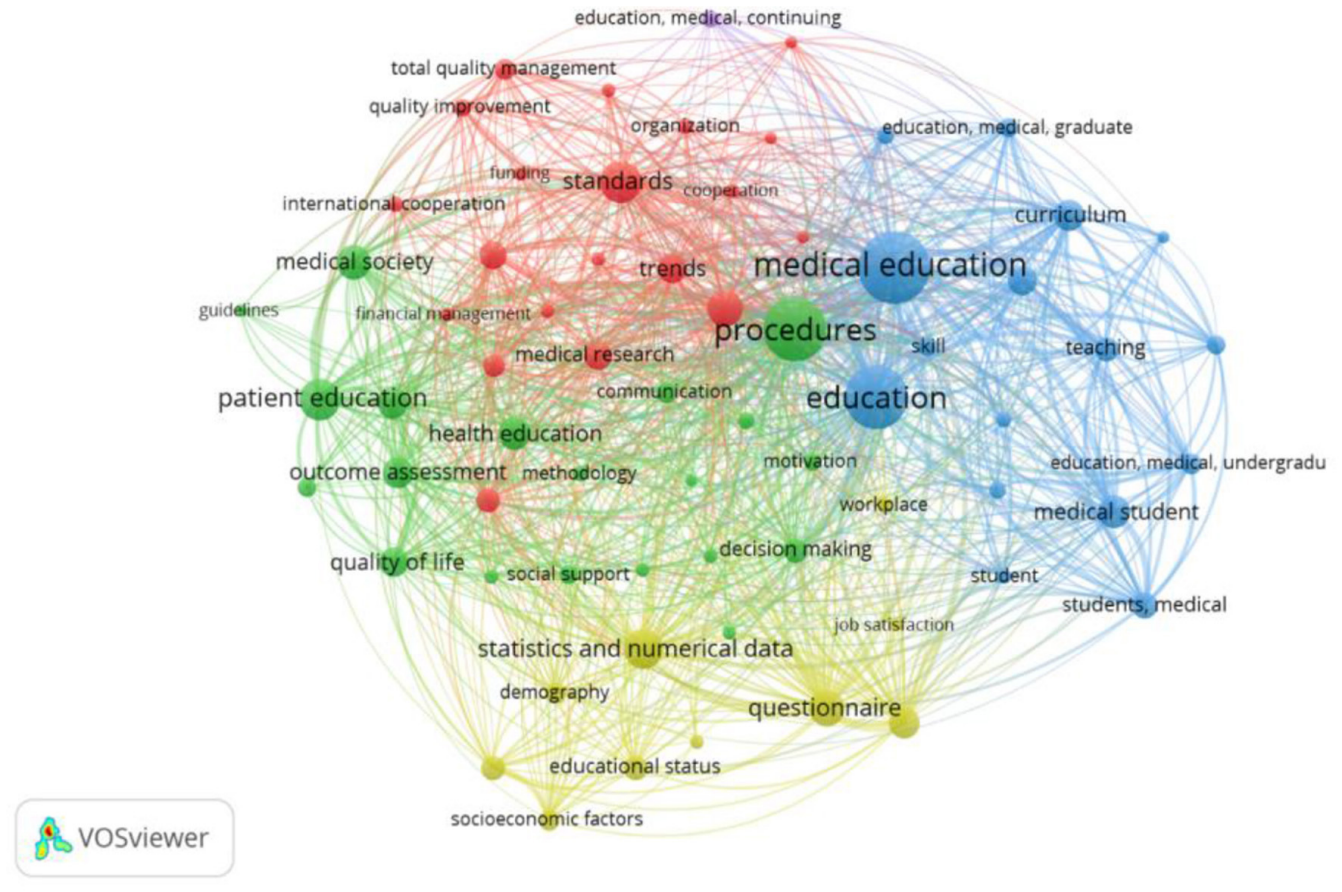
Bibliometric analysis of the key phrase “medical education”.

**Figure 2:**
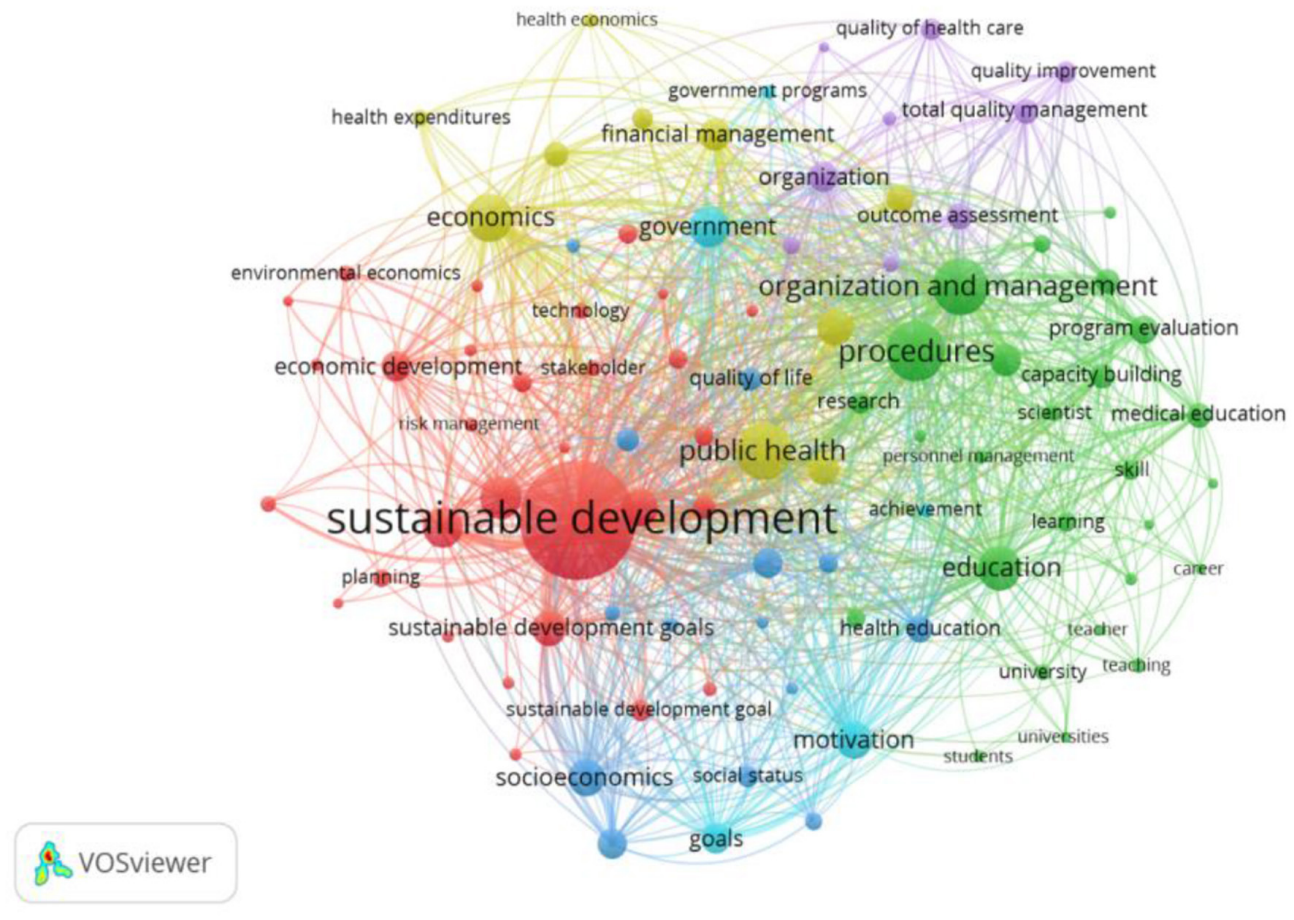
Bibliometric analysis of the key phrase “sustainable development goals”.

The data of bibliometric analysis of the keyword “medical education” states that various aspects of quality assurance are an integral part of the medical education analysis (Figure 1, tool—VOSviewer, analyzed 109,000 documents in all areas, selected the most cited 2,000 for the period 2016–2020, the minimum number of mentions of keywords: 25, excluding keywords related to specialized medical terminology). Interestingly, a significant number of keywords presented in Figure 1 are related to Sustainable Development Goals (Figure 2, tool–VOSviewer, analyzed 8,000 documents in the field of “medicine” for the period 2016–2020, the minimum number of mentions of keywords: 50, excluding keywords related to specialized medical terminology), which determines the additional relevance of this study.

Furthermore, the analysis was performed on data related to life expectancy and healthy life expectancy indices and indicators of the Universitas 21 rating leaders. The analysis involved constructing a table of relevant indicators and examining the top 10 countries in the ranking of Universitas 21 and the first 36 countries in the ranking and their respective indicators in the Life Expectancy Index 2020 and the WHO Healthy Life Expectancy Index 2018. The analysis aimed to explore the relationship between these variables and determine whether there is a significant correlation or associ ation between them. We utilized a simple linear regression model and interpretation of its coefficients to illustrate the interconnection between said indices. For the first round of analysis, we selected the Life Expectancy Index 2020 rating as the dependent variable, with the national educational Universitas 21 ranking as an independent variable. The second round of analysis accounted for data from the WHO Healthy Life Expectancy Index 2018 as a dependent variable and Universitas 21 ranking as an independent variable.

Success (S) in achieving results in any activity depends on two components: skill (M) and luck (L). This approach is reflected in Michael Mauboussin's success equation (53), which is a weighted linear function of skill and luck:


(1) $$\begin{array}{l} S\;=\;aM+bL, \end{array}$$

where the value a reflects the relative weight of skill in the range from 0 to 1, and as a result, b = 1 – a.

Suppose we relay this approach to assessing education quality, in that case, we encounter the term “luck” as a very limited set, which is reduced to success not in achieving the quality of the educational program, but luck regarding the contingent involved in the current educational program. Furthermore, all the success, which is directly related to the educational program quality, can be measured by skill, and a value in (1) is around one. Thus, we can only talk partly about the paradox of skill in the case of the “education quality” dynamic system. For the system “education quality”, the term “skill” in (1) has three components:

1. internal quality assurance of the educational program q.i.
2. mobility in improving the educational program in response to the challenges mo.
3. external evaluation by stakeholders and independent agencies q.e.

However, the success of the university's educational activities depends not only on skill and (at a minimum) luck but also on a third parameter—the authority of the university (R), which is determined, inter alia, by world rankings. Thus, the equation of Mauboussin's success can be upgraded to the following form:


(2) $$\begin{array}{l} S=aM+bL+cR \end{array}$$

where a + b + c = 1.

Considering components of the skill defined above, Eq. (2) will take on the form


(3) $$\begin{array}{l} S=a\left(QI+Mo+QE\right)+bL+cR. \end{array}$$

External evaluation of the education quality by stakeholders and independent agencies is one of the most effective ways to determine the competitiveness level of an educational program. This tool is effective in cases when quality assessment indicators are clearly defined.

A multi-factor model for assessing the quality of medical education is proposed, which is based on the view from the “inside” (quality indicators of the components of the educational program and the quality assurance system) and “from the outside” (indicators of the success of the educational program according to the evaluation methods of the Ministry of Education and Science, the National Agency for Higher Education Quality Assurance).

## 2. Materials and methods

Since rating agencies, in addition to general university rankings, also implement university rankings by area (which actually assess educational program(s) quality within a field), additional mechanisms for educational program benchmarking are emerging.

Given the similar nature of the external and rating university evaluation, the success in Eq. (2) for the university can be interpreted as follows:


(4) $$\begin{array}{l} S\;=\;EXT+INT, \end{array}$$

where


(5) $$\begin{array}{l} EXT=aQE+bL+cR. \end{array}$$

(6) $$\begin{array}{l} INT=\;a\left(QI+M\right). \end{array}$$

The success of the educational program is also determined by Eq. (4). However, given the insignificance of luck's contribution to educational program quality, equation (5) takes the form


(7) $$\begin{array}{l} EXT=aQE+cR. \end{array}$$

In this study, we try to describe the component EXT in (7), considering both the rating agencies' requirements for education quality assessment by area and experience of external accreditation of educational programs by the National Agency for Higher Education Quality Assurance (Ukraine) in 2020–2021. We focus on determining the indicators QE and R because the weight factors a and c may vary for a particular evaluation period.

## 3. Results

The presence of Ukrainian institutions providing higher medical education in the world rankings is currently a future matter. Advances on the path to the entry of Ukrainian universities to the branch medical ratings are possible due to benchmarking the main rating indicators and understanding the landscape and depth of their content. In addition, one should consider that industry rankings do not assess specific programs but the whole subject area. This fact determines the additional relevance of developing procedures aimed at the evaluation of educational programs. An attempt to evaluate medical education and the institutions that provide it was made in the ranking of the top 100 faculties of domestic universities (Top best faculties of Ukraine according to Forbes, 2021) and the ranking of the top 200 universities in Ukraine (HEI rating “TOP-200 Ukraine 2021”, 2021). Indirectly, these ratings can be used to assess educational programs' quality, but they have a low level of detail in the assessment as their main drawback.

Assessment of educational institutions' quality is also carried out according to the scientific component (54). However, it is more likely to be named an outcome of quality education, expressed in the level of implementation of research results. Such attestation is currently implemented by the Ministry of Education and Science of Ukraine for subordinated universities, which does not allow us to compare the results with the indicators of universities subordinated to the Ministry of Health of Ukraine. In addition, this tool does not allow the allocation of a separate sector “medicine,” as it evaluates the activities of universities in the field of “Biology and Health.”

Based on the assessment of certain university performance indicators, the Ministry of Education and Science of Ukraine has developed a methodology for allocating state budget expenditures (55). Since 1 January 2021, this method is also applicable to universities subordinated to the Ministry of Health of Ukraine. Among other indicators, this technique uses the indicator of international recognition and the indicator of graduate employment, which are part of the second term of Eq. (7).

Using the data of ratings of the Life Expectancy Index 2020, the WHO Healthy Life Expectancy Index 2018, and Universitas 21: Ranking of National Higher Education Systems 2020, we constructed a table of relevant indicators of Universitas 21 rating leaders related to life expectancy and healthy life expectancy. Table 1 presents the top 10 countries in the ranking of Universitas 21 plus Ukraine. For a more detailed analysis of the relationship between the ratings, the first 36 countries of the Universitas 21 ranking and their respective indicators in the Life Expectancy Index 2020 and the WHO Healthy Life Expectancy Index 2018 were considered.

**Table 1:** The Life Expectancy Index 2020 and the WHO Healthy Life Expectancy Index 2018 for the first 10 countries in Universitas 21 rating plus Ukraine.

**Country/rating**	**Universitas 21: ranking of national higher education systems 2020**		**Life Expectancy Index 2020**		**World Health Organization: Healthy Life Expectancy Index 2018**			
	**Rating**	**Index**	**Rating**	**Index (yrs)**	**Rating**	**Index(yrs)**		
						**Mean**	**Male**	**Female**
The USA	1	100.0	37	78.9	40	68.5	66.9	70.1
Switzerland	2	90.1	3	83.8	4	73.5	72.4	74.5
Denmark	3	85.7	30	80.9	24	71.8	70.7	73.0
Singapore	4	84.5	4	83.6	1	76.2	74.7	77.6
Sweden	5	84.3	11	82.8	17	72.4	71.5	73.4
The United Kingdom	6	83.6	27	81.3	23	71.9	70.9	72.9
Canada	7	83.2	15	82.4	7	73.2	72.0	74.3
Finland	8	82.8	22	81.9	25	71.7	69.8	73.5
Australia	9	82.2	7	83.4	9	73.0	71.8	74.1
The Netherlands	10	81.6	17	82.3	20	72.1	71.3	72.8
Ukraine	36	47.8	114	72.1	100	64.0	60.3	67.6

**Figure 3:**
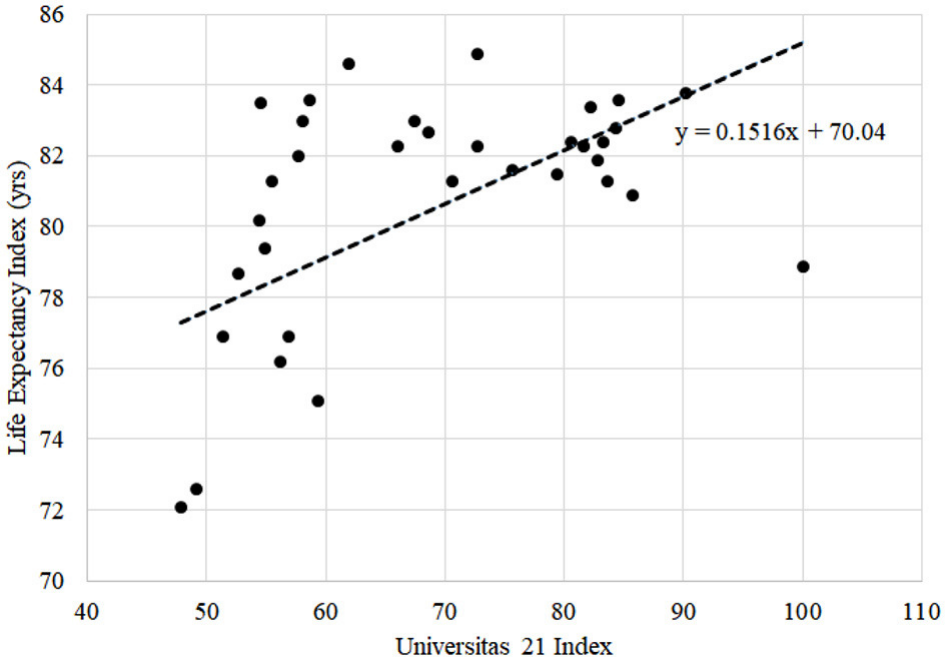
Universitas 21 index against Life Expectancy Index (in years).

Figure 3 demonstrates the results of the life expectancy assessment depending on the national level of higher education. The coefficient at *x* in the linear regression equation can be interpreted as follows: with an increase of the Universitas 21 index by one unit, the value of the Life Expectancy Index increases by ~0.152 years. This fact allows us to conclude that education (primarily medical) quality directly impacts the parameters used in compiling the Life Expectancy Index. However, the model is limited and illustrative, as it does not consider several factors directly related to healthcare and health services.

**Figure 4:**
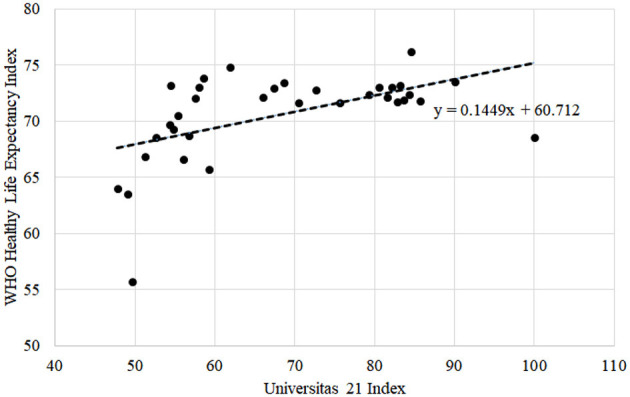
Universitas 21 index against the WHO Healthy Life Expectancy Index (in years).

Figure 4 is similar to Figure 3, but it estimates the life expectancy according to the WHO Healthy Life Expectancy Index 2018 relative to the Universitas 21. The coefficient at *x* in the linear regression equation for this case shows that by increasing Universitas 21 by one unit, the value of life expectancy according to the WHO Healthy Life Expectancy Index increases by ~0.145 years. This dependence is also not decisive but should be noted in the context of medical education analysis.

Table 2 presents European universities ranking in the “Medicine” field according to QS World University Rankings. The university's rank is determined by the value of “Overall Score”. It is important to consider that the positions of compared universities in the ranking may differ significantly despite slight differences in the value of the “Overall Score.” In the general case, the assessment of university performance according to the QS methodology (56) is based on six key factors:

- Academic Reputation (40%)
- Employer Reputation (10%)
- Faculty/Student Ratio (20%)
- Citations per Faculty (20%)
- International Faculty Ratio (5%)
- International Student Ratio (5%)

**Table 2:** QS rankings of Europe universities by subject “medicine”.

**Rank**	**University**	**Overall score**	**H-index citations**	**Citations per Paper**	**Academic reputation**	**Employer reputation**
2	The University of Oxford	96.4	96.3	97.2	96.4	94.2
4	The University of Cambridge	94.1	91.4	93.9	96	94.2
6	Karolinska Institutet	92.3	94.1	92.1	96	73.2
9	UCL	91.3	94.3	94.3	88	82.3
10	Imperial College London	90.8	93.1	96.9	87.1	84.4
17	King's College London	87.6	91.6	92.8	85.2	74.5
21	The University of Edinburgh	86.4	90.3	95.2	81.7	73.6
23	London School of Hygiene & Tropical Medicine	86.2	89.3	95.9	83.3	65.7
30	Ruprecht- Karl-Universität Heidelberg	84.2	91.2	91.4	78.6	71.3
32	The University of Amsterdam	84	90.9	94.1	77.8	66.5

This study has an interest in analyzing the data related to the EXT component in (6) in the field of “Medicine”. Therefore, we use QS World University Rankings by Subject, which takes into account the following indicators:

- Academic Reputation
- Employer Reputation
- Citations per paper
- H-index

Indicators “Academic Reputation” and “Employer Reputation” are based on the data obtained during surveys of academic institutions' employees and employers, respectively. Indicators “Citations per paper” and “H-index” are used to measure the scientific productivity of the university by subject. The principles and reasons for using these indicators are described in detail in (57). The weights of mentioned indicators differ by area, hence within the “Medicine” subject, the following set is used: Academic Reputation, 40%; Employer Reputation, 10%; Citations per Paper, 25%; and H-index, 25%. For universities training health professionals, one can see that priority is given to reputation in academia and scientific productivity, while reputation among employers clearly recedes into the background.

We propose using QS World University Rankings by Subject in the model discussed in this study: Academic Reputation and Employer Reputation are included in the component *cA* of Eq. (6), and Citations per Paper and H-index are accounted for by the component *aQE*. Weight factors for these indicators should be consistent with the weight factors of the other components in terms of Eq. (7). However, the QS Rankings approach provides a general idea of prioritization in university performance indicators in the “Medicine” subject framework.

The methodology for external quality assurance of educational programs incorporated by the National Agency for Higher Education Quality Assurance has the following features [the description is based on the regulatory framework of the National Agency (58)]:

Accreditation is carried out by nine (for the bachelor and master level of higher education) or 10 (for the phd level of higher education) criteria:

- Criterion 1. Project and goals of the educational program.
- Criterion 2. Structure and content of the educational program.
- Criterion 3. Access to educational programs and education results acknowledgment.
- Criterion 4. Learning and teaching within the educational program.
- Criterion 5. Control measures, student evaluation, and academic integrity.
- Criterion 6. Human resources.
- Criterion 7. The learning environment and material resources.
- Criterion 8. Internal quality assurance of the educational program.
- Criterion 9. Transparency and publicity.
- Criterion 10. Learning through research (for PhD level, not considered in the current study).

Each criterion evaluates the educational program and educational activities within the program according to the evaluation scale, which covers four levels of compliance.

- Level “A”: the educational program and activities within the program fully meet the assessed criteria and have an innovative/exemplary character.
- Level “B”: the educational program and educational activities within the program generally meet the assessed criterion with shortcomings that are not significant.
- Level “E”: the educational program and/or educational activities within the program generally do not meet the assessed criterion, but identified shortcomings can be eliminated within 1 year;
- Level “F”: the educational program and/or educational activities within the program do not meet the assessed criteria. The identified shortcomings are fundamental and/or cannot be eliminated within 1 year.

The educational program can receive one of four grades:

- Accredited and marked as exemplary (A).
- Accredited (B).
- Conditionally accredited (E).
- Rejected in accreditation (F).

The following conditions for determining accreditation grade are proposed based on the number of corresponding criteria grades:


(8) $$\begin{array}{c} A:A\geq 5,\;E=0,\;F=0; \end{array}$$

(9) $$\begin{array}{c} B:B>5,\;E=0,\;F=0; \end{array}$$

(10) $$\begin{array}{c} E:E\leq 2; \end{array}$$

(11) $$\begin{array}{c} F:E>2\;and/or\;F>0. \end{array}$$

This study analyzes the accreditation results of more than 110 educational programs in 2020 and 8 months of 2021 within the direction 22 “Medicine” (according to the national classification of fields of knowledge) (state and private medical universities).

The above evaluation criteria have been considered in previous studies (59–65) as those that have a significant impact on the quality of educational programs in the process of external expertise.

Table 3 is constructed on the basis of the abovementioned 110 programs' accreditation data. For each educational program and corresponding evaluation criteria, we present the ratio “Success/Innovation”.

**Table 3:** Educational program accreditation results within the direction 22 “medicine”.

**[-50,11.8]7646mm Program Criterion**	**Success/innovation**								
	**C1**	**C2**	**C3**	**C4**	**C5**	**C6**	**C7**	**C8**	**C9**
221 ≪Stomatology≫	100/20	100/0	100/12	96/4	100/16	96/28	96/24	100/8	100/24
222 ≪Medicine≫	92/4	73/0	100/12	96/0	92/4	96/15	100/8	96/4	100/8
223 ≪Nursing≫	100/0	75/0	100/0	100/0	100/0	83/8	100/0	100/8	100/12
224 ≪Technologies of Medical Diagnostics and Treatment≫	100/0	100/0	100/0	100/0	100/0	100/0	100/0	100/25	100/0
226 ≪Pharmacy≫	86/9	86/0	100/0	86/0	100/0	86/14	90/14	95/14	95/0
227 ≪Physical Rehabilitation≫	94/13	88/0	100/0	94/0	100/0	94/6	100/6	100/6	100/19
228 ≪Pediatrics≫	100/25	100/25	100/0	100/0	100/0	100/25	100/25	100/50	100/25
229 ≪Public Health≫	100/29	100/14	100/14	100/0	100/14	86/43	100/29	100/43	100/14

Success (*k*_*s*_) is the percentage of grades A (*n*_*A*_) and B (*n*_*B*_) of the total accreditations number in the program for each area (*N*) and relevant criteria. Innovation (*k*_*i*_) is the percentage of grades A (*n*_*A*_) of the total accreditations number in the program for each area (*N*) and relevant criteria.


(12) $$\begin{array}{c} k_{s}=\frac{n_{A}+n_{B}}{N}\cdot 100\% \end{array}$$

(13) $$\begin{array}{c} k_{i}=\frac{n_{A}}{N}\cdot 100\% \end{array}$$

The criteria were combined into meaning-based complexes for a more effective and simple assessment of the success and innovation of educational activities within the educational program and the whole direction. “Content and Potential” (CP) complex combines criteria 1, 2, and 7; “Algorithms” (A) complex combines criteria 3 and 5; and “Personalities” complex combines criteria 4, 6, 8, and 9. Further evaluation of these complexes allows for a comprehensive assessment of both the educational program and the set of programs. Table 4 contains the Success/Innovation ratio calculations for the described criteria complexes for educational programs of 22 “Medicine” direction.

**Table 4:** Evaluation of success/innovation of criteria complexes.

**Program**	**Success (** ***k***_**s**_ **)/innovation (** ***k***_**i**_ **)**		
	**Complex CP (C1, C2, and C7)**	**Complex A (C3 and C5)**	**Complex P (C4, C6, C8, and C9)**
221 Stomatology	98.7/14.7	100/14	98/16
222 Medicine	88.3/4	96/8	94/6.8
223 Nursing	91.7/0	100/0	95.8/7
224 Technologies of Medical Diagnostics and Treatment	100/0	100/0	100/6.25
226 Pharmacy	87.3/7.7	100/0	90.5/7
227 Physical Rehabilitation	94/6.3	100/0	97/15.5
228 Pediatrics	100/25	100/0	100/25
229 Public Health	100/24	100/14	96.5/25

Success (*k*_*s*_) and innovation (*k*_*i*_) are included in the *QE* component of Eq. (7), as they pose a convenient and accessible tool for external quality analysis. Equation (7) in explicit form, adapted to assess educational activities quality is:


(14) $$\begin{array}{c} EXT=aQE+cR=a\left(1+\frac{\ln\Phi_{e}}{\ln\Phi_{b}}+\overset{3}{\underset{j=1}{\sum }}\frac{k_{ij}}{k_{sj}}\right)\\ +c\left(1+\overset{n}{\underset{k=1}{\sum }}{r_{gen}}_{k}+\overset{n}{\underset{m=1}{\sum }}{r_{loc}}_{m}\right) \end{array}$$

The component *QE* presenting an external quality evaluation of educational activities consists of the following components:

Φ_*b*_ is the component determined by the basic funding from the Ministry of Education and Science. In the case of university-level assessment, this component is formed for the whole institution.

Φ_*e*_ is the component determined by funding from external sources (except for basic funding): basic funding of science, grants, economic contracts, governmentally funded research projects, and special funds of the Ministry of Education and Science, e.g., $\Phi_{1}^{R\&D}+\Phi_{2}^{grants}+\Phi_{3}^{economic\;contracts}+\Phi_{4}^{other\;sources}$. In the case of university-level assessment, this component is formed separately for each area.

Ratio $\frac{\ln\Phi_{e}}{\ln\Phi_{b}}$ illustrates the financial independence of the educational institution. The ratio of logarithms allows effective handling of cases where funding from external sources significantly exceeds the basic funding and vice versa. Maximum value $\frac{\ln\Phi_{e}}{\ln\Phi_{b}}$ for *F*_*e*_≫Φ_*b*_ is limited by 2.

$\overset{3}{\underset{j=1}{\sum }}\frac{k_{ij}}{k_{sj}}$ is the component formed by the sum of Success/Innovation ratios for each direction and the relevant criteria complexes. In the case under consideration, the criteria are divided into three complexes, so the maximum value of the sum is 3.

University authority level R depends on two components, the first of which (*r*_*gen*_) characterizes the university's position in the rankings (international and/or national) as a whole institution. The second component *r*_*loc*_ characterizes the direction's position in ratings by area. The maximum value for both components is 1, while the second component allows accounting for situations when the rating by area does not exist or the university is not included in such rating. In these situations, it is recommended to set an indicator $\overset{n}{\underset{m=1}{\sum }}{r_{oc}}_{m}$ individually in each evaluation process for internal authority level assessment and to use the recommended parameters for external evaluations:

- $\overset{n}{\underset{m=1}{\sum }}{r_{loc}}_{m}=0,1$, if the university is not included in the ranking by area
- $\overset{n}{\underset{m=1}{\sum }}{r_{loc}}_{m}=0,3$, if the rating by direction does not exist.

## 4. Discussion

To improve the quality of educational activities, it is proposed to allocate contact points for the application of additional funding within each set of evaluation criteria:

1. “content and potential” complex (criteria 1, 2, and 7)
  - Bonuses for educational program project working groups.
  - Forming of additional budget for the engagement of external specialists in designated areas.
  - Constant refreshing of material assets, technical learning instruments, purchase of additional literature and/or online services subscriptions, etc.

2. “algorithms” complex (criteria 3 and 5)
  - Additional funding is aimed at building a pipeline of constant communication with external stakeholders to synchronize learning results and gained competencies with the required expertise in the field.
  - Funding for building and facilitating internal systems of academic integrity in accordance with the national regulatory framework and best international practices.

3. “personalities” complex (criteria 4, 6, 8, and 9)
  - Allocating the budget aimed to form a bonus system for learning process stakeholders and an academic mobility system independent from international programs.
  - Ensuring the “lifelong learning study” concept by funding constant professional growth of human resources associated with the learning process.
  - Funding of internal quality assurance system for educational activities and synchronizing it with best practices in the field.
  - Establishing open catalogs of educational offers, programs, and resources along with expenditures for creating and supporting media resources.

Funding of proposed contact points must be performed according to sources defined by the first component of Eq. (14). Each component in the numerator of the ratio $\frac{\ln\;F_{e}}{\ln\;F_{b}}$ can ensure funding distribution via separate directions requiring financial support as mentioned above.

We propose to overlook the pipeline of funding sources allocation for the university considering perspective marking of meeting criteria as exemplary for each external quality assurance of the educational program:

1. Identifying shortcomings for defined criteria.
2. Considering the possibility of increasing criterion completion level due to additional funding.
3. Selecting financial source(s) present in the university which can fund (in the framework of the current regulatory base) chosen activities aimed at criterion completion level increase.
4. Funding of certain activities or development of infrastructure through chosen financing source.

**Figure 5:**
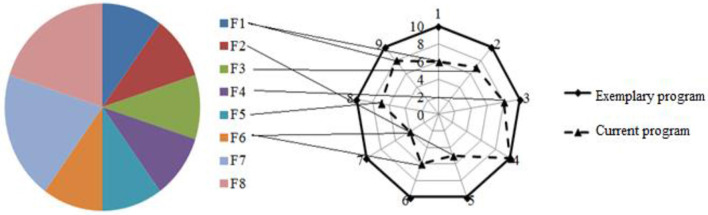
Definition of funding sources for university activity assuming perfect meeting of external quality assurance criteria.

Figure 5 illustrates the definition of funding sources for university activity. In this approach, we propose to assume the perfect meeting of external quality assurance criteria considering criteria 1–9 for educational programs. Here, F1–F8 are funding sources for university activities: F1 and F2 are incomes from domestic and foreign students, F3 and F4 are incomes from scientific activities from domestic and foreign clients, F5 and F6 are incomes from grant-related activities from domestic and foreign donors, F7 is an income from additional educational offers, and F8 is an income from additional non-educational offers. Criteria grades and percentages of funding sources are presented on a pie diagram as a demonstration.

**Figure 6:**
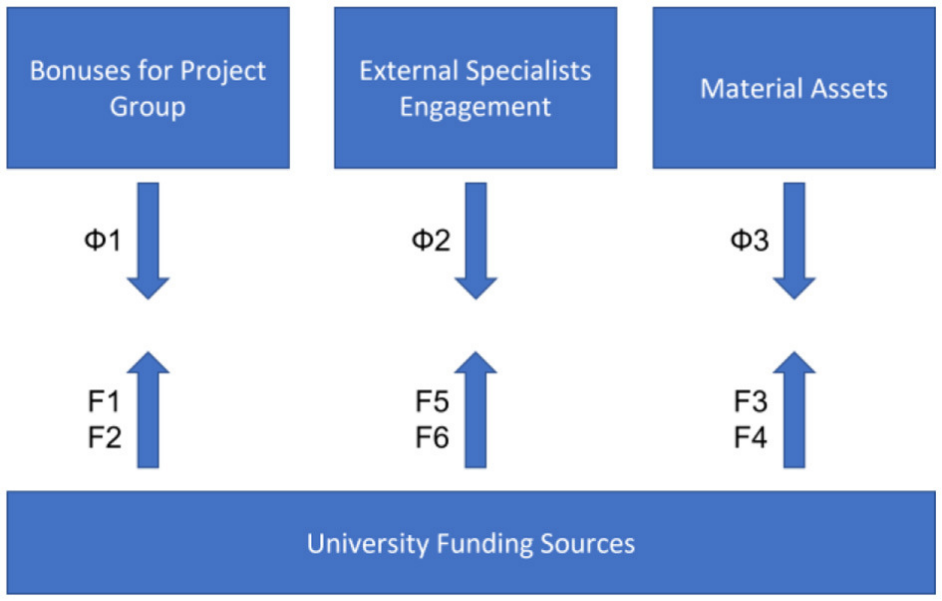
Algorithm for selecting funding source for the “Content and Potential”: Φ is the necessary funding volume, and Fi is the university funding sources (number of sources corresponds to Figure 5).

This approach not only diversifies expenditure sources but also can act as a catalyst for the diversification of university income sources through various types of educational, research, and production activities. Diversification of income sources becomes urgent since each source allows funding specific activities following the income conditions (estimates defining future expenses). Increasing revenue streams of different origins covers a broader range of tasks that need funding. For example, we propose the algorithm for selecting funding sources for the “Content and Potential” complex (criteria 1, 2, 7) as shown in Figure 6.

The proposed method of external quality assessment has advantages over analogs, which are given in the literature review:

1. the methodology takes into account independent indicators obtained by third parties.
2. the methodology can evaluate the indicators numerically.
3. the methodology allows for a retrospective analysis of progress (positive or negative) in ensuring the quality of the educational program.

## 5. Conclusion

External accreditation and ratings are the most effective tools for quality assessment. However, ratings often neglect the field of study with rare exceptions when the rating has a direction selection option. In addition, some rankings require data input from the university, which universities do not always want to provide and hence are dropped from the rankings. In this case, the rating cannot be considered a full and independent assessment of the university but can take part in the overall assessment tool construction. Therefore, the external assessment of education quality through accreditation expertise comes to the fore.

Mauboussin's success equation requires modification for educational program quality assessment to evaluate the luck level at the design stage and add a component of external assessment by stakeholders that can be done through indicators of educational ratings. The paradox of skill, in which, other things being equal, the one who took advantage of the coincidence is more successful, in this case, can be interpreted as an opportunity to minimize risks. Risks here are feedback from external stakeholders and competitive or tender principles for obtaining funding from external sources.

The proposed approach to the formation of the external quality evaluation algorithm of educational activities *EXT* covers both the indicators that form the component of the external evaluation by stakeholders and independent agencies and the reputation component R formed by the university authority. We considered boundary cases for all algorithm components and provided examples of success and innovation calculation for the 22 “Medicine” subject.

The discussed analysis method of external evaluation results proposed in the study can be effectively scaled to obtain conclusions at three levels: program, university (within the benchmarking of general university components of the educational program), and national (within the benchmarking of similar programs).

The methodology presented in this study allows us to assess the educational program quality both qualitatively and quantitatively.

According to the proposed method, an important calculation result is the determination of external quality assessment criteria requiring additional funding. It is possible to establish the relationship between current financing from external sources (excluding basic funding, which is also obtained in the competition for indicators defined by the relevant formula) and the state of implementing certain external evaluation criteria and drawing conclusions about strengthening activity in specific areas.

## Data availability statement

The original contributions presented in the study are included in the article/supplementary material, further inquiries can be directed to the corresponding author.

## Author contributions

Conceptualization and writing—original draft preparation: AA, IV, and SL. Methodology, project administration, and software: AA and IV. Validation: AA, IV, and BG. Formal analysis: SB, AA, and SL. Investigation: AA, IV, SL, and SB. Data curation: IV and TM. Writing—review and editing: AA, IV, SL, BG, and TM. Visualization: IV and BG. Supervision: AA. Funding acquisition: TM. All authors contributed to the article and approved the submitted version.

## References

[1] Vercellini P Viganò P Somigliana E. Government should monitor quality of medical education. BMJ (Online). (2015) 350:h2937. 10.1136/bmj.h2937 26036865

[2] Cook DA Reed DA. Appraising the quality of medical education research methods: the medical education research study quality instrument and the newcastle-ottawa scale-education. Acad Med. (2015) 90:1067–76. 10.1097/ACM.0000000000000786 26107881

[3] Molano PA. Proposal of high-quality accreditation standards for undergraduate programmes in medicine with a focus on primary health care in Colombia. Educ Medica. (2022) 23:100731. 10.1016/j.edumed.2022.100731

[4] Mazroie S Dehghani MR Yamani N Sabzevari S. Evaluation of medical university deputies' and managers' perspectives on the outcomes of Institutional Accreditation of Medical Universities from 2018-2019. Stride Dev Med Educ. (2021) 18:e10406. 10.22062/sdme.2021.195647.1040

[5] Taghavinia M Maleki M Arabshahi K. Educational leadership in education development centers: a qualitative study. J Educ Health Promot. (2021) 10:46. 10.4103/jehp.jehp_733_20 34084793 PMC8057170

[6] Torralba KMD Katz JD. Quality of medical care begins with quality of medical education. Clin Rheumatol. (2020) 39:617–8. 10.1007/s10067-019-04902-w 31902030

[7] Domínguez LC. Tools for the evaluation of learning climate in posgraduate training: Synthesis of evidence in the light of psychometric definitions. Educacion Medica. (2018) 19:335–49.

[8] Blouin D Tekian A Kamin C Harris IB. The impact of accreditation on medical schools' processes. Med Educ. (2018) 52:182–91. 10.1111/medu.13461 29044652

[9] Da Dalt L Callegaro S Mazzi A Scipioni A Lago P Chiozza ML. A model of quality assurance and quality improvement for post-graduate medical education in Europe. Med Teacher. (2010) 32:e57–e64. 10.3109/01421590903199734 20163217

[10] Hasan T. Doctors or technicians: Assessing quality of medical education. Adv Med Educ Pract. (2010) 1:25–9. 10.2147/AMEP.S13877 23745059 PMC3643128

[11] Blouin D Tekian A. Accreditation of medical education programs: moving from student outcomes to continuous quality improvement measures. Acad Med. (2018) 93:377–83. 10.1097/ACM.0000000000001835 28746072

[12] Nahla N. University-company collaboration: what are the obstacles in Algeria? SocioEcon Challen. (2023) 7:59–64. 10.21272/sec.7(1).59-64.2023

[13] Soltes M Gavurova B. Quantification and comparison of avoidable mortality - causal realtions and modification of concepts. Technol Econ Dev Econ. (2015) 21:917–38. 10.3846/20294913.2015.1106421

[14] Yu Y Xinxin W Ruoxi L Tingting Y. The mediating role of human capital in the relationship between education expenditure and science and technology innovation: evidence from China. Socio Econ Challen. (2023) 7:129–38. 10.21272/sec.7(1).129-138.2023

[15] Artyukhov A Volk I Vasylieva T Lyeonov S. The role of the university in achieving SDGs 4 and 7: a Ukrainian case. E3S Web Conf. (2021) 250:04006. 10.1051/e3sconf/202125004006

[16] Petrushenko Y Vadym A Vorontsova A Ponomarenko O. Sustainable development goals as a tool for strategic planning in communities: a bibliometric analysis of research. E3S Web Conf. (03005) 2020:202. 10.1051/e3sconf/202020203005

[17] Matos L Kasztelnik K. Transformational educational leadership and the innovative strategies engaging online faculty for the excellent teaching performance in the United States. Bus Ethics Leadersh. (2021) 5:6–21. 10.21272/bel.5(1).6-21.2021

[18] Dzwigoł H. Leadership in the research: determinants of quality, standards and best practices. Bus Ethics Leadersh. (2021) 5:45–56. 10.21272/bel.5(1).45-56.2021

[19] Šoltés M Gavurova B. Identification of the functionality level of day surgery in Slovakia. Ekon Cas. (2014) 62:1031−51.

[20] Skrynnyk O Vasilieva T. Comparison of Open Learning Forms in Organizational Education. Cagliari: CEUR Workshop Proc. p. 1314–1328. Available online at: https://ceur-ws.org/Vol-2732/20201314.pdf

[21] Francis C. Accreditation of Study Programs on Addictions in Nigerian Universities: Challenges, Opportunities, and the Need for Advocacy. Adiktologie. (2022) 22:27–33.

[22] Pavlenko O Martynets V Dreval O Smolennikov D. Analysis of influence of the quality of specialist training on social and economic development. Calitatea Acces Success. (2020) 21:81–6.

[23] Lyeonov S Liuta O. Actual problems of finance teaching in ukraine in the post-crisis period. In:Azarmi T Amann W, editors. The Financial Crisis. Cham: Springer International Publishing. (2016) p. 145–152. 10.1007/978-3-319-20588-5_8

[24] Martins FP Cezarino LO Liboni LB Botelho Junior AB Hunter T. Interdisciplinarity-based sustainability framework for management education. Sustainability. (2022) 14:12289. 10.3390/su141912289

[25] Kvitka S Starushenko G Koval V Deforzh H Prokopenko O. Marketing of Ukrainian higher educational institutions representation based on modeling of webometrics ranking. Mark Manag Innov. (2019) 5:60–72. 10.21272/mmi.2019.3-05

[26] Savga L Krykliy O Kyrychenko K. The role of internal and external stakeholders in higher education system in Ukraine. Bus Ethics Leadersh. (2018) 2:32–43. 10.21272/bel.2(1).32-43.2018

[27] Zborník TS Miovský M. Comprehensive evaluation of the online lifelong education course prevention and treatment of substance use disorders for physicians in the czech republic: study protocol. Adiktologie. (2021) 21:179–83.

[28] Skliar I. Towards the assurance of transparency and quality of higher education in Ukraine: national qualification framework. Bus Ethics Leadersh. (2018) 2:96–105. 10.21272/bel.2(1).96-105.2018

[29] Miovsky M Volfova A Johnson K Peters R Koutsenok I Heaps M. New trends in education and training programs in addictions at the higher education and university levels: what kind of specialised programs are trending? Adiktologie. (2021) 21:201–9.

[30] Zuluaga-Ortiz R DelaHoz-Dominguez E Camelo-Guarín A. Academic efficiency of engineering university degrees and its driving factors. A PLS-DEA Approach J Int Stud. (2022) 15:107–21. 10.14254/2071-8330.2022/15-2/8

[31] Istenič Starčič A Lebeničnik M. Investigation of University 'students' perceptions of their educators as role models and designers of digitalized curricula. Hum Technol. (2020) 16:55–91. 10.17011/ht/urn.20200224216328862900

[32] Costa F Figueira-Cardoso S. University Outreach, indigenous knowledge, and education: a project with the Pataxó in Brazil. Eur J Interdiscip Stud. (2022) 14:39–55. 10.24818/ejis.2022.03

[33] Jankelová N. Entrepreneurial orientation, trust, job autonomy and team connectivity: implications for organizational innovativeness. Eng Econ. (2022) 33:264–74. 10.5755/j01.ee.33.3.28269

[34] Gad S Yousif NBA. Public management in the education sphere: prospects for realizing human capital in the development of knowledge management technologies. Adm SI Manag Public. (2021) 10:151–72. 10.24818/amp/2021.37-10

[35] Stuchlý P Virágh R Hallová M Šilerová E. CRM and its importance for business competitiveness. Agris -Line Pap Econ Inform. (2020) 12:93–8. 10.7160/aol.2020.120108

[36] Delibasic M Ivanis M Pupavac D Shilina M. Modeling citizens satisfaction with higher education: a case study of Rijeka. Montenegrin J Econ. (2022) 12:18. 10.14254/1800-5845/2022.18-4.12

[37] Draskovic V Jovovic R Rychlik J. Perceptions of the declining quality of higher education in the selected SEE countries. J Int Stud. (2020) 13:286–94. 10.14254/2071-8330.2020/13-4/20

[38] Dzionek-Kozlowska J Neneman J. Are economic majors “indoctrinated” by their education? Public good game quasi-experiment. Econ Sociol. (2022) 15:110–24. 10.14254/2071-789X.2022/15-2/7

[39] Caballero-Morales SO Cordero-Guridi JJ Alvarez-Tamayo RI. Cuautle-Gutiérrez L. Education 40 to support entrepreneurship, social development and education in emerging economies. Int J Entrepreneurial Knowl. (2020) 8:89–100. 10.37335/ijek.v8i2.11935843246

[40] Vlasov M Panikarova S Draskovic M. Evaluating university academic efficacy: institutional approach. Montenegrin J Econ. (2020) 16:241–50. 10.14254/1800-5845/2020.16-1.16

[41] Hitka M Štarchon P Lorincová S Caha Z. Education as a key in career building. J Bus Econ Manag. (2021) 22:1065–83. 10.3846/jbem.2021.15399

[42] Wach K Bilan S. Public support and administration barriers towards entrepreneurial intentions of students in Poland. Adm SI Manag Public. (2021) 4:67–80. 10.24818/amp/2021.36-04

[43] Moreno-Carmona C Feria-Domínguez JM Merinero-Rodríguez R. Are University management teams strategic stakeholders within higher education institutions? A clinical study. Econ Sociol. (2022) 15:141–59. 10.14254/2071-789X.2022/15-1/9

[44] Gavurova B Tucek D Kovac V. Economic aspects of public procurement parameters in tertiary education sector. Adm Si Manag Public. (2019). 10.24818/amp/2019.32-04

[45] Krisnaresanti A Julialevi KO Naufalin LR Dinanti A. Analysis of entrepreneurship education in creating new entrepreneurs. Int J Entrepreneurial Knowl. (2020) 8:67–76. 10.37335/ijek.v8i2.112

[46] Abdimomynova A. Entrepreneurship education prospects in the public-private partnership system. Montenegrin J Econ. (2021) 17:83–92. 10.14254/1800-5845/2021.17-2.7

[47] Barrientos-Báez A Martínez-González JA García-Rodríguez FJ Gómez Galán J. Entrepreneurial competence perceived by university students: quantitative and descriptive analysis. J Int Stud. (2022) 15:40–9. 10.14254/2071-8330.2022/15-2/334235236

[48] Bauters M Pejoska J Durall E Saarikivi K Wikström V Falcon M. Are you there? Presence in collaborative distance. Work Hum Technol. (2021) 17:261–93. 10.14254/1795-6889.2021.17-3.5

[49] Kumar V Nayak KP Bhinder HS. The technology acceptance model and learning management system: a study on undergraduate tourism and hospitality students. Eur J Interdiscip Stud. (2021) 13:65–89.

[50] Prívara A Kiner A. Immigrant employment in the slovak hospitality industry: profiles, experience, and education. J Tour Serv. (2020) 20:167–82. 10.29036/jots.v11i21.223

[51] Tercanli H Jongbloed B. A systematic review of the literature on living labs in higher education institutions: potentials and constraints. Sustainability. (2022) 14:12234. 10.3390/su141912234

[52] Kibrit G Altinay F Dagli G Altinay Z Sharma R Shadiev R. Evaluation of sustainability and accessibility strategies in vocational education training. Sustainability. (2022) 14:12061. 10.3390/su141912061

[53] Mauboussin MJ. The Success Equation: Untangling Skill and Luck in Business, Sports, and Investing. Boston: Harvard Business Review Press.

[54] Order of Ministry of Education and Science About the Results of Governmental Attestation of Higher Education Institutes Regarding Their Scientific (Scientific-Technical) Activities. (2021).

[55] Decree Decree of Cabinet of Ministers of Ukraine About State Budget Expenditures Distribution amongst Higher Education Institutions Based on Educational Scientific and International Activity Indicators. (2019).

[56] Q.S. World University Rankings—Methodology. Q.S. Top Univ. Website.

[57] Q.S. World University Rankings by Subject: Methodology. Q.S. Top Univ. Website.

[58] Order of Ministry of Education and Science About Approval of Guidelines for Accreditation of Educational Programs Used for Student Training. (2019).

[59] Yu Y. Performance analysis of public investment in chinese university education based on regional differences and influencing factors. Busi Ethics Leader. (2023) 7:37–49.

[60] Nezai A Ramli M Refafa B. The relationship between the scientific activities in research laboratories with webometrics ranking of algerian universities: an empirical investigation. Busi Ethics Leader. (2022) 6:67–82. 10.21272/bel.6(1).67-82.2022

[61] Vidic F. Knowledge asset as competitive resource. Socio Econ Challen. (2022) 6:8–20. 10.21272/sec.6(4).8-20.2022

[62] Safarov Q Sadiqova S Urazayeva M. Methodological approach to identification of innovative determinants of human capital management. Mark Manag Innov. (2022) 2:255–67. 10.21272/mmi.2022.2-23

[63] MacNeil P Khare A Jugdev K. International inequity patterns in youth and young adults related to COVID-19: advancing sustainable development goals on well-being, education, and employment. Health Econ Manag Rev. (2022) 3:60–72. 10.21272/hem.2022.3-06

[64] Smiianov VA Vasilyeva TA Chygryn OY Rubanov PM Mayboroda TM. Socio-economic patterns of labor market functioning in the public health: challenges connected with covid—19. Wiadomosci Lekarskie (Warsaw, Poland : 1960). (2020) 73:2181–7. 10.36740/WLek202010114 33310944

[65] Vasylieva T Gavurova B Dotsenko T Bilan S Strzelec M Khouri S. The behavioral and social dimension of the public health system of european countries: descriptive, canonical, and factor analysis. Int J Envir Res Public Health. (2023) 20:5 10.3390/ijerph20054419 36901427 PMC10002141

